# Induction and Subversion of Human Protective Immunity: Contrasting Influenza and Respiratory Syncytial Virus

**DOI:** 10.3389/fimmu.2018.00323

**Published:** 2018-03-02

**Authors:** Stephanie Ascough, Suzanna Paterson, Christopher Chiu

**Affiliations:** ^1^Section of Infectious Diseases and Immunity, Imperial College London, London, United Kingdom

**Keywords:** respiratory syncytial virus, influenza, respiratory disease, CD4^+^ T cell, B cell, innate immunity, toll-like receptor, RIG-I like receptor

## Abstract

Respiratory syncytial virus (RSV) and influenza are among the most important causes of severe respiratory disease worldwide. Despite the clinical need, barriers to developing reliably effective vaccines against these viruses have remained firmly in place for decades. Overcoming these hurdles requires better understanding of human immunity and the strategies by which these pathogens evade it. Although superficially similar, the virology and host response to RSV and influenza are strikingly distinct. Influenza induces robust strain-specific immunity following natural infection, although protection by current vaccines is short-lived. In contrast, even strain-specific protection is incomplete after RSV and there are currently no licensed RSV vaccines. Although animal models have been critical for developing a fundamental understanding of antiviral immunity, extrapolating to human disease has been problematic. It is only with recent translational advances (such as controlled human infection models and high-dimensional technologies) that the mechanisms responsible for differences in protection against RSV compared to influenza have begun to be elucidated in the human context. Influenza infection elicits high-affinity IgA in the respiratory tract and virus-specific IgG, which correlates with protection. Long-lived influenza-specific T cells have also been shown to ameliorate disease. This robust immunity promotes rapid emergence of antigenic variants leading to immune escape. RSV differs markedly, as reinfection with similar strains occurs despite natural infection inducing high levels of antibody against conserved antigens. The immunomodulatory mechanisms of RSV are thus highly effective in inhibiting long-term protection, with disturbance of type I interferon signaling, antigen presentation and chemokine-induced inflammation possibly all contributing. These lead to widespread effects on adaptive immunity with impaired B cell memory and reduced T cell generation and functionality. Here, we discuss the differences in clinical outcome and immune response following influenza and RSV. Specifically, we focus on differences in their recognition by innate immunity; the strategies used by each virus to evade these early immune responses; and effects across the innate-adaptive interface that may prevent long-lived memory generation. Thus, by comparing these globally important pathogens, we highlight mechanisms by which optimal antiviral immunity may be better induced and discuss the potential for these insights to inform novel vaccines.

## Introduction

Acute respiratory tract infection is a leading cause of morbidity and mortality worldwide, 2.7 million deaths having been attributed to lower respiratory tract infections (LRTI) in 2013 (1). Recent mortality statistics for England and Wales indicate that influenza and pneumonia were among the principal causes of death among both under 4 year olds and adults aged over 80 years (2), while in the US, pneumonia is known to be a predominant cause of hospitalization among pediatric and elderly populations, accounting for 36 hospital stays per 10,000 people (3). The causes of both upper and lower respiratory tract illnesses can frequently be traced to viral infections. A recent analysis of community-acquired childhood pneumonia in the US found that two thirds of patients with a detectable pathogen were infected with one or more viruses, of which the most common was respiratory syncytial virus (RSV) (4). This is consistent with global mortality data, which indicates that two of the most important causes of pneumonia in under 5 years olds are RSV and influenza (1). During the first 2 years of life, all children will have been infected at least once with RSV, with approximately two thirds infected by the end of their first year; in the UK this translates to a peak risk of hospital admission at approximately 1 month of age (5). With increasing age, subsequent RSV episodes decrease in severity, with a mild-to-moderate upper respiratory tract infection (URTI) in healthy adults. However, in elderly patients RSV once again becomes a cause of severe disease (6). Unlike RSV, influenza shows a less dramatic bias toward pediatric infections, and the majority of seasonal influenza-related deaths occur amongst elderly patients (7–9), although in a USA study, Thompson et al. found that rates of influenza-related hospitalization among infants and young children were on a par with those in the over-65s (8). This indicates that each year both pathogens represent an enormous disease burden in vulnerable population groups.

Influenza and RSV both circulate seasonally, with annual outbreaks in temperate regions occurring during winter months, although in tropical climates the seasonality is less distinct and peaks of infection occur year round (10). Both pathogens, which are responsible for recurrent infections throughout life, are enveloped single-stranded negative-sense RNA viruses. Influenza belongs to the Orthomyxoviridae family, and unlike RSV, which belongs to the Paramyxoviridae family, the genomes of the influenza genus are divided into segments. This segmented nature aids the emergence of variant viruses through antigenic shift (by genome segment reassortment), which in combination with antigenic drift (by accumulated point mutation), has led to enormous genetic diversity. Naturally occurring infection by influenza viruses is known to induce long-lasting protective immunity, although this is strain-specific. Recurrent infections therefore occur due to the antigenic variation seen in circulating strains over time. In contrast, reinfection with antigenically similar strains of RSV occurs frequently throughout life, especially in children and the elderly, indicating that the immunity induced by natural infection provides little long-term protection against reinfection (11). The natural histories of these two viruses are a consequence of the divergent immune sequelae that they provoke, but the lack of persistently protective immunity following natural infection in each case can be seen as two sides of the same coin. The continued lack of a universal, effective vaccine against either virus means that both continue to cause huge burdens of mortality and morbidity across the globe.

## Clinical, Epidemiological, and Virological Features of RSV

**Figure 1:**
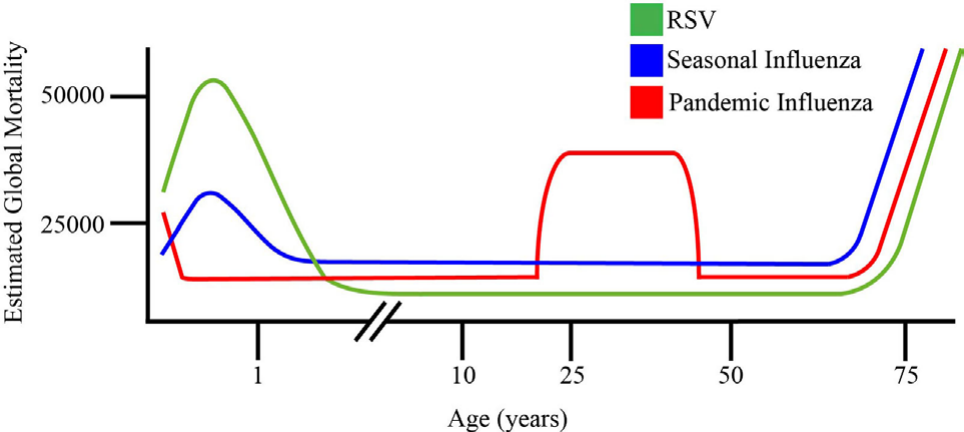
Schematic representation of comparative age distribution of respiratory syncytial virus (RSV), 2009 pandemic influenza and seasonal influenza mortality. RSV is a leading viral cause of infant death (8), with maternal antibodies offering temporally constrained protection to neonates against seasonal flu and RSV. This is limited in the case of novel pandemic influenza strains not previously encountered by the maternal immune system. Young adults are disproportionately affected by pandemic influenza strains, relative to seasonal influenza and RSV. Pandemic and seasonal influenza, as well as RSV all cause dramatic mortality in elderly individuals, reflecting the age-related decline of immune function in this population (8, 19).

Respiratory syncytial virus causes a spectrum of illness, which ranges from mild URTIs, or otitis media, to severe LRTIs, encompassing bronchiolitis. Bronchiolitis is associated with the infiltration of inflammatory cells into the airways, over-production of mucus, and edema of the respiratory tract (12). This leads to a progressive narrowing of the airways with increased airflow obstruction, causing tachypnea and hypoxia. The characteristic airway inflammation and epithelial necrosis associated with RSV-induced bronchiolitis has also been implicated in long-term impairment of lung function, as RSV infection during childhood is believed to be an important risk factor for the development of asthma and allergic wheeze during later life (13, 14). Indeed, Blanken et al. have shown that treatment with the anti-RSV monoclonal antibody (mAb) palivizimab, reduces postviral wheeze at 1 year of age (15). An estimated global burden of 33.1 million acute respiratory tract infections, resulting in 3.2 million hospital admissions in under 5 year olds, have been attributed to RSV (16). Approximately one third of deaths resulting from acute lower respiratory infection in the first year of life can be ascribed to RSV, with between 94,600 and 149,400 childhood deaths related to RSV every year (17, 18). However, with limitations in healthcare provision in low and middle income countries, accurate epidemiological assessment in many regions is difficult due to inconsistent diagnostics and under-reporting of cases. In addition to the pediatric disease burden, RSV also contributes to significant morbidity and mortality in the elderly, immunocompromised individuals, and those with pulmonary or cardiac comorbidities (see Figure 1) (6).

The 15.2 kb genome of RSV consists of 10 genes coding for 11 proteins, many of which have been targeted in attempts to induce pathogen-specific immunity. Eight of the proteins are internal and include the matrix (M) and two non-structural proteins (NS1 and NS2). Further internal proteins are all associated with the construction of the nucleocapsid. These include the products of the M2 gene, which are two polypeptides resulting from overlapping open reading frames: a nucleocapsid-associated transcription factor (M2-1) and the associated polypeptide involved in genome replication (M2-2). The nucleoprotein (N) and phosphoprotein (P) together interact with the RNA-dependent polymerase (L) to form the nucleocapsid. The 3 remaining transmembrane proteins are found in the viral envelope: the small hydrophobic protein (SH) which enhances membrane permeability in host cells through ion channel formation (20); the highly glycosylated major attachment protein (G), which mediates viral attachment to the host cell; and the fusion protein (F), which mediates both virus-to-cell and cell-to-cell fusion. RSV is relatively antigenically stable compared to influenza and only the G protein undergoes extensive variation akin to hemagglutinin (HA) and neuraminidase (NA) found on the surface of influenza. This has led to the classification of circulating RSV strains into two different antigenic groups (A or B) based upon divergent sequences within the G protein ectodomain. Furthermore, in addition to its genetic diversity, the virus also expresses G protein in a secreted form, which may function as an antigenic decoy (21).

The F protein has presented an attractive target for rational vaccine design since the early days of RSV vaccine development. While both G and F proteins are immunogenic targets of the antibody response, F is the only one essential for cell entry and (unlike G) is highly conserved across all known RSV strains. However, the F protein presents an unusual target for the immune system, as the metastable prefusion conformation present in the virus rapidly undergoes a number of structural changes both spontaneously and following host cell binding that results in a secondary, more thermodynamically stable postfusion form (22–24). Both forms of this protein display epitopes but only three of the antigenic sites (I, II, and IV) found on the prefusion protein are preserved following conformational change. These have been defined by mAbs, with site I recognized by mAbs such as 2F, 44F, or 45F and site IV by mAb19 and 101F (25, 26). Site II is targeted by the humanized monoclonal IgG antibody, palivizumab, currently the only clinically approved RSV immunoprophylaxis in use. In high-risk infants, monthly administration of palivizumab reduces RSV hospitalizations by at least 50%, showing that systemic IgG alone can confer protection against LRTI. However, the relatively modest efficacy, high cost and requirement for repeated dosing precludes its use in resource-constrained settings. Furthermore, all of the antibodies raised against these antigenic sites show at best moderate neutralizing activity and postfusion F protein-based vaccines have so far failed to demonstrate efficacy in clinical trials (27, 28). In contrast, the most potent RSV neutralizing antibodies are raised against the site Ø epitope, which is found exclusively in the pre-fusion conformation of the protein, the structure of which was only recently elucidated. The mAbs D25, AM22, and 5C4, which target this site, have a neutralizing potency up to 100-fold greater than palivizumab, giving rise to the possibility of more effective antibody-mediated protection if site Ø can be preferentially induced (24, 29).

While vaccination remains the most cost-effective health intervention for infectious diseases, there are currently no effective vaccines available against RSV. This underdeveloped state of vaccine development is due in part to the unenviable position that an early RSV vaccine candidate has in the history of vaccines. During the late 1960s, a clinical trial involving vaccination of children with an alum-precipitated formalin-inactivated RSV vaccine preparation (FI-RSV) led to exacerbated disease upon subsequent natural infection with live RSV. Although almost half (43%) of the infants receiving the vaccine displayed at least a fourfold rise in serum neutralizing antibody titer and almost all (91%) had at least a fourfold rise in complement fixing antibody, 80% of vaccinees required hospitalization for pneumonia and bronchiolitis on subsequent RSV exposure, resulting in two fatalities (30, 31). Considerable research effort over the decades since has sought to determine the mechanisms responsible for this immunopotentiation or “vaccine-enhanced disease.” Subsequent studies have suggested that formalin-inactivation led to the deformation of B cell epitopes, which may have promoted the development of non-neutralizing antibodies (32). This in combination with CD4^+^ T cell responses biased toward production of Th2-associated interleukins (IL) (IL-4, IL-5, and IL-13), may have exaggerated immunopathology upon later infection to RSV (33). There is also recent evidence that deficient toll-like receptor (TLR) stimulation resulting in a lack of antibody affinity maturation may have played a role (34).

The state of RSV vaccine development has drastically improved over the last decade in view of increased understanding of disease burden and antigenic targets. Almost 50 vaccine and mAb products are in development, of which several are currently in clinical trials (35, 36). However, the recent failure of a recombinant F protein nanoparticle vaccine candidate in a phase III trial of elderly adults despite showing immunogenicity in earlier clinical trials (37–40), demonstrates that there is still an urgent need to improve our understanding of immune correlates of protection for this pathogen.

## Clinical, Epidemiological, and Virological Features of Influenza

Acute respiratory infections with seasonal influenza cause an estimated 3–5 million cases of severe illness and 250,000–500,000 deaths globally each year (17, 41), with an accumulated cost in the USA alone of ~$87 billion per annum (42). In addition to this ever present threat to public health, there is the constant possibility that a novel virus with a high case-fatality rate may emerge and prove capable of initiating a pandemic. Over the past 100 years there have been four such pandemics: the H1N1 Spanish flu in 1918 which was responsible for 50–100 million deaths; the H2N2 outbreak in 1957 which caused 100,000 deaths; the H3N2 outbreak in 1968 which caused 700,000 deaths; and the recent H1N1 pandemic in 2009 which claimed over 15,000 lives (43). Although large variations have been seen in the excess mortality caused by successive pandemic influenza strains, these appear to disproportionately affect young adults relative to seasonal influenza and RSV (Figure 1), these deaths have been linked to an influenza initiated “cytokine storm” and a susceptibility to lethal secondary bacterial pneumonia (44, 45). Influenza viruses which have their origins within the avian viral reservoir have accounted disproportionately for many of these deaths, and continue to represent an emerging threat to human health as the progenitors of the next influenza pandemic. Currently, two strains of highly pathogenic avian influenza are circulating in birds and causing sporadic outbreaks in humans (H7N9 in South East Asia, and the panzootic H5N1), both of which have pandemic potential.

As with RSV, influenza viruses enter hosts either intranasally or less commonly *via* the eye, following exposure to infected secretions. Influenza infection is then initiated within the airway by the attachment of HA to sialic acid receptors on the surface of the host epithelium. While RSV is uniquely adapted to human cells, with attachment thought to be mediated by the chemokine receptor CX3CR1 (46), HA may be adapted to a number of species and specificity is thought to be a critical factor in host tropism. Avian influenza HA preferentially binds to α(2,3)-sialic acid linkages, while influenza viruses circulating in humans possess HA subtypes that recognize and attach to the α(2,6)-sialic acid linkages more commonly expressed in the human respiratory tract. It is possible to modify this binding specificity through the mutation of a single amino acid within the receptor binding domain, increasing the likelihood of the virus acquiring the capability to infect a new host species. This is of particular concern in pigs and certain birds, such as turkeys, which have both α-2,3 and α-2,6 linkages, and are thus capable of acting as mixing vessels to generate reassortant viruses (47).

Influenza viruses are divided into A, B, and C types. Influenza A viruses, which are the pathogens responsible for the majority of seasonal and all pandemic influenza infections, infect a range of mammals and birds, while types B and C typically infect humans. They all possess segmented genomes: influenza A and B contain eight RNA segments and influenza C seven. The influenza A genome encodes 11 core and accessory viral proteins. A further two proteins (negative sense protein and the N-terminal truncated variant N40) may have a role in late-stage infection but as yet their functions remain unclear (48, 49). In common with RSV there are two non-structural proteins (NS1 and NS2) and influenza also possesses two matrix proteins; M1 is found within the lipid bilayer surrounding the virus core and M2 is a transmembrane ion channel. The internal core of the virus is a ribonucleprotein RNA-dependent polymerase complex composed of a nucleoprotein (NP), polymerase acidic (PA), and two polymerase basic subunits (PB1 and PB2) along with an alternatively transcribed proapoptotic peptide, PB1-F2. Influenza viruses are divided into subtypes based on sequence variations in their main surface glycoproteins: HA (which is divided into two subunits, HA1 and HA2) and NA. These are involved in host cell attachment and host cell egress, respectively. Thus far, 18 different HAs and 11 NAs have been defined.

In common with RSV, the surface glycoproteins of influenza are the major targets of the protective humoral response. However, unlike RSV, both proteins are apt to vary greatly as a result of antigenic drift and shift (50). In comparison, the genes encoding the internal virus proteins such as the M gene, are highly conserved between influenza A viruses (50). While it is possible to generate effective vaccines which offer protective immunity against circulating strains, the changing nature of both seasonal and pandemic viruses means that individuals need to be vaccinated repeatedly as mutations are gathered and it is not possible to produce a tailored vaccine quickly and affordably in the quantities required to respond to a novel pandemic virus.

## Innate Immunity Following Respiratory Virus Infection

### Intrinsic and Immediate Barriers to Infection in the Respiratory Tract

During the initial stages of respiratory virus infection the naso- and oro-pharynx are the primary sites of exposure. These areas are covered by a thick mucus layer, which protects the epithelial cells. However, mucin macromolecules and sialic acid compounds found within this mucus throughout the respiratory tract can be cleaved by influenza NA, leaving HA free to bind to cell-surface sialic acid residues and initiate viral entry (51). The action of NA has been shown to be crucial in this regard, as oseltamivir blocks influenza infection of mucus-producing human bronchial epithelial cells (52). RSV lacks this sialic-acid cleaving capacity and host cells respond to infection by expression of mucins, the specific mucin protein MUC5AC is elevated in A549 human alveolar epithelial (53) and bronchial epithelial cells (54). MUC5AC is also linked to the innate immune response, being elevated through chemokine (C-X-C motif) receptor CXCR2 signaling in a mouse model of RSV infection (55). Although the exact extent to which mucus upregulation alters infection risk is unclear in humans, the protective effects of MUC5AC overexpression during influenza infection of mouse models (56) suggest that this often overlooked first line of defense plays an important role in warding off both respiratory viruses. Within mucus, other immediate mechanisms including cationic host defense peptides such as cathelicidin have also been shown to play a role in disrupting respiratory viruses at the point of virus encounter (57, 58). Both influenza and RSV envelopes are directly damaged by cathelicidin, which represents one of a number of ancient defense mechanisms that may act immediately to prevent entry of virus into nasal cells. Emerging evidence suggests that respiratory viruses may harness posttranslational modification of these defenses to abrogate their function. Indeed, it remains to be seen whether these early barrier mechanisms can be manipulated for the purposes of prophylaxis and for the time being the focus is therefore on the innate-adaptive immune axis for vaccine development.

### The Role of Pattern Recognition Receptors in Respiratory Virus Infection

Airway epithelial cells, neutrophils, alveolar macrophages (AMs) and dendritic cells (DCs), and members of the innate lymphoid cell (ILC) family resident in the respiratory tract make up the first line of innate immune cellular defense. These cells are capable of sensing viral RNA, the predominant pathogen-associated molecular pattern (PAMP) of influenza and RSV, as well as cell-generated danger-associated molecular patterns (DAMPs), through several families of highly conserved pattern recognition receptors (PRRs). Innate recognition through PRRs has a profound impact on the entire immune response and it is increasingly obvious that pathogens interfere with these pathways to modulate both short and long-term immunity.

**Figure 2:**
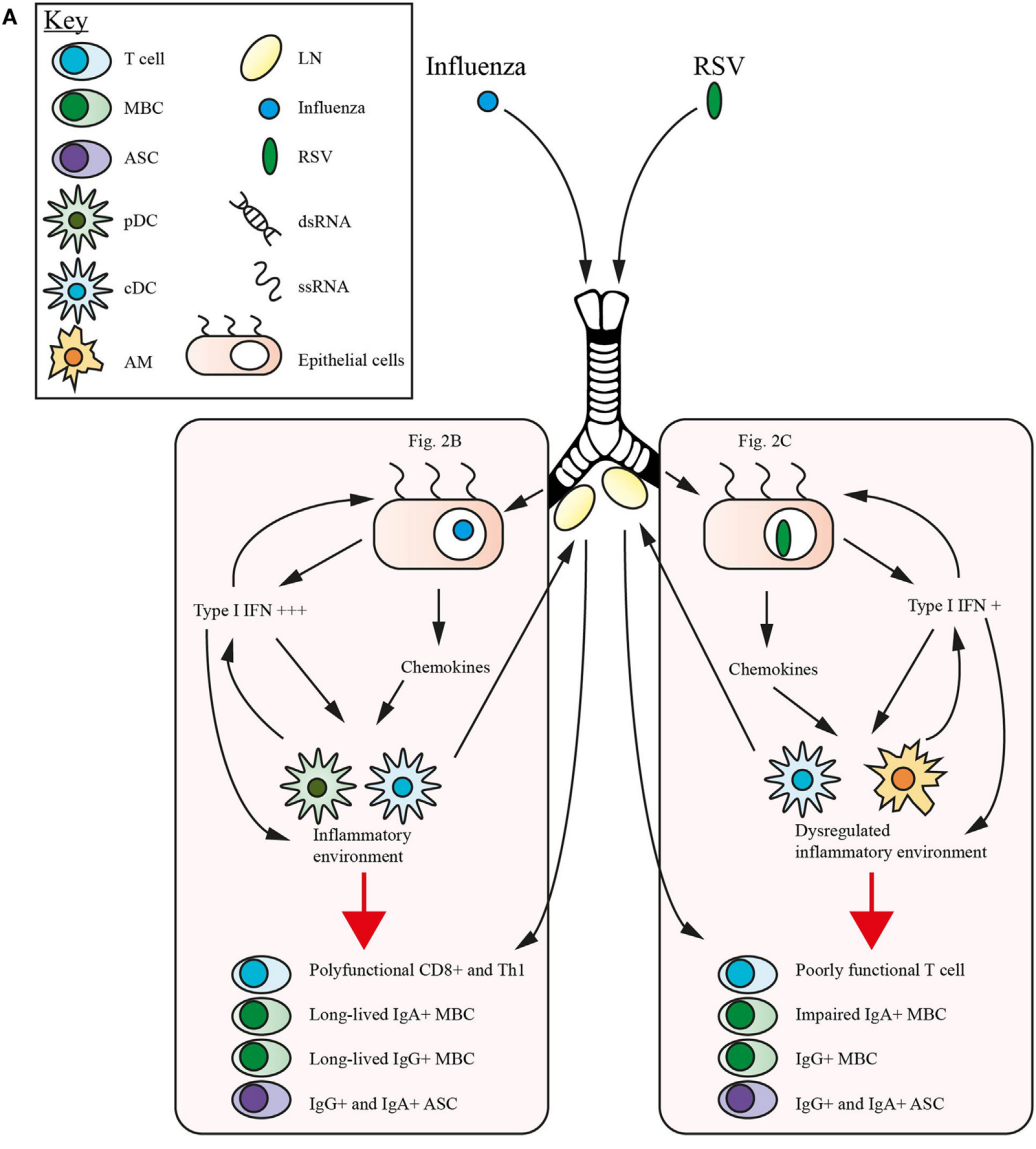

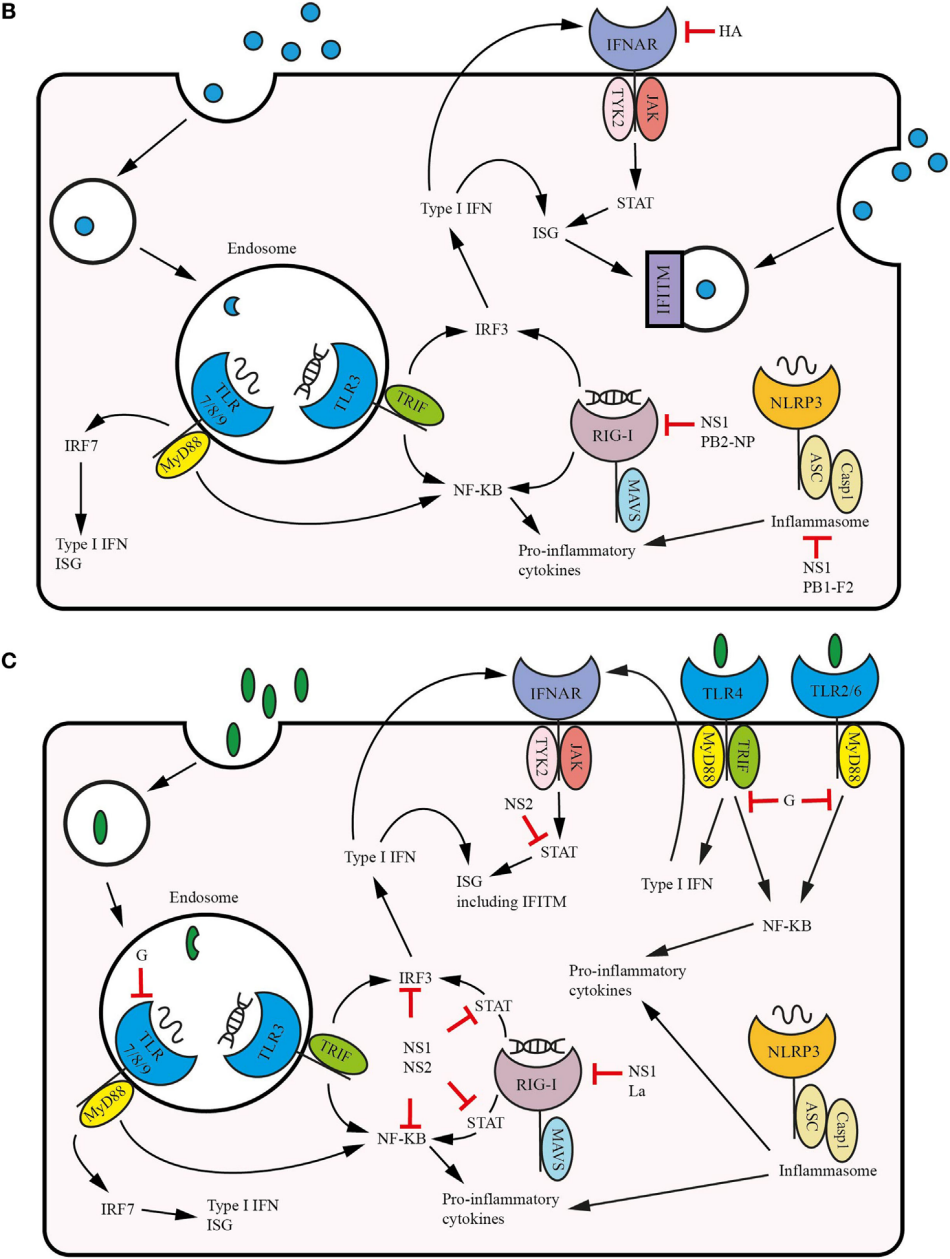
Comparative immunity in respiratory syncytial virus (RSV) and influenza. Within the mucosal surfaces of the lung the host immune response to each virus feature intersecting and non-overlapping traits. **(A)** Influenza and RSV establish infection in the lung epithelial cells, initiating the release of type 1 interferons (IFNs) which act in a feedback loop along with release of proinflammatory cytokines such as interleukin (IL)-1β and IL-18, and upregulation of IFN-stimulated genes (ISGs) to promote an inflammatory environment. This inflammatory milieu, which in influenza recruits conventional dendritic cells (cDCs) and plasmacytoid DCs (pDCs) as the primary type 1 IFN producing antigen-presenting cells (APCs), promotes optimal signaling and memory generation. This includes polyfunctional CD8^+^ and Th1 T cells, and immunocompetent IgG^+^ and IgA^+^ ASCs and memory B cells (MBCs). Following viral clearance T cells remain in the lung as T resident memory (Trm) and MBCs and T cells traffic into the systemic compartment. RSV infection of the lung follows a broadly similar pattern, although the primary type 1 IFN producing APCs are alveolar macrophage (AM) and cDCs. The lower levels of type 1 IFN produced during infection impact the generation of memory responses with poorly functional T cells, skewing toward Th2/Th17 generation in early life and a profound defect in the induction of an IgA^+^ MBC subset. As the influenza virus establishes infection within a lung epithelial cell **(B)** it enters the endosome and following viral uncoating it triggers TLR3 signaling with double-stranded RNA (dsRNA) and TLR 7/8/9 with single-stranded RNA (ssRNA), in addition retinoic acid inducible gene-I (RIG-I) and the inflammasome are stimulated by contact with dsRNA and ssRNA, respectively. These signaling pathways individually stimulate nuclear factor-κB (NF-κB), IRFs and upregulate ISGs and the production of type 1 IFNs. These mediators are released by the cell and recognized by IFNα receptor (IFNAR), leading to positive feedback, which also stimulates the sequestration of virus by IFN-induced transmembrane protein (IFITM), preventing viral spread. Influenza acts to mitigate the actions of the pattern recognition receptors (PRRs), especially RIG-I and the inflammasome through NS1-mediated inhibition, while hemagglutinin (HA) has recently been found to trigger ubiquitination of IFNα receptor (IFNAR), downregulating IFNAR1 expression. In the case of RSV infection within a lung epithelial cell **(C)** the action of the intracellular PRRs is augmented by the engagement of the extracellular TLRs 4/2/6. RSV strategically targets signaling pathways at multiple points, most notably the action of G protein upon TLR7/8/9 and TLRs 4/2/6, and NS1 upon a number of pathways, particularly STAT signaling.

In Figure 2, we highlight the mechanisms involved in the induction of innate and adaptive immunity to influenza and RSV, and how the disparity in protection may be related to the manner in which these pathogens engage PRRs.

The three key families of PRRs involved in sensing viral infection are TLRs, retinoic acid inducible gene-I (RIG-I), and nucleotide-binding oligomerization domain (NOD)-like receptors (NLRs). These function as critical components of the initial innate immune response, governing the release of cytokines including the type I interferons α and β (IFN α/β). This was recently shown by Forero et al., who found by transcriptomic analysis of human nasal epithelial cell cultures that the innate response to viral infections such as influenza is driven by type I and III IFN (59). This was also associated with induction of the chemokine (C-X-C motif) ligands for CXCR3; CXCL9, CXCL10, and CXCL11, indicating a coordinating role for the early innate epithelial response in the recruitment of monocytes and generation of effector and memory CD8^+^ T cells (59). Large scale genetic studies have also demonstrated that susceptibility to RSV-induced bronchiolitis are associated with polymorphisms in innate immune genes (60, 61). However, the contribution of individual genetics to innate immune responses (both positive and negative) is yet to be clearly delineated. A recent study of common single-nucleotide polymorphisms in RLRs and IL-4 signaling genes have showed no link to susceptibility to severe RSV infection and there remains controversy in the field (62). Nevertheless, it is clear that innate recognition of PAMPs and DAMPs triggers the induction of the antiviral immune response and might reasonably be a point at which viruses attempt to evade detection.

#### Intracellular Toll-Like Receptors Recognizing RSV and Influenza

The intracellular receptors TLR3, TLR7, TLR8, and TLR9 all play a role in sensing viral infection. TLR3 is an important activator of type I IFNs; interacting with the toll/IL-1 receptor (TIR) domain-containing adaptor protein inducing IFN beta (TRIF) to activate serine-threonine kinases IκKε and TANK-binding kinase 1 (TBK1), phosphorylating the IFN regulatory factor 3 (IRF3) which induces the expression of IFNβ. TLR3 recognizes double-stranded RNA (dsRNA), a potent DAMP, which may be released from virus-infected phagocytosed cells (63). Within human respiratory epithelial cells, which constitutively express TLR3, downstream proinflammatory cytokine production may be protective, but can also lead to pathology (64). This is borne out by animal studies which indicate that, despite an elevated viral load, *tlr3*^−/−^ mice show decreased infiltration of inflammatory cells into the lungs and increased survival rates following lethal influenza challenge (65). Conversely, in a *tlr3*^−/−^ mouse model of RSV infection, alteration of the immune environment leads to increased IL-13 and an associated overproduction of mucus (66). In this context, it is interesting that both Influenza A and RSV infection lead to upregulated expression of TLR3 in pulmonary epithelial cells but that this may result in different outcomes (67, 68). Adding further to this complexity, influenza and RSV infection can promote increased production of CXCL8/IL-8 upon subsequent re-stimulation with TLR3 ligands, suggesting that previous virus exposure can induce “trained” immunity that may be both beneficial and harmful to the host (67, 68). However, despite the importance of TLR3 in the early immune response, *tlr3*^−/−^ mice have been found to generate normal humoral and T cell responses to sublethal influenza infection (69), which suggests that, at least in the case of influenza, TLR3 stimulation is not essential for the induction of adaptive immunity.

This redundancy can also be seen in the activation of TLR7; neither activation of TLR3 nor TLR7 alone appear capable of stimulating IFN-mediated antiviral defense during infection. TLR7 is highly expressed in plasmacytoid dendritic cells (pDCs), being capable of detecting the single stranded RNA (ssRNA) present in viruses which are taken up into the endosome. It interacts with the adaptor protein, myeloid differentiation factor 88 (MyD88) (70, 71) leading to the activation of nuclear factor-κB (NF-κB) and IRF7, transcription factors that are responsible for stimulating the expression of proinflammatory cytokines and type I IFNs (72–74). Murine DCs deficient in either TLR7 or MyD88 show reduced levels of IFNα induction following influenza virus infection (70, 71). This is in accordance with animal models of infection, which found that naive *myd88*^−/−^ *tlr3*^−/−^ and *tlr7*^−/−^ mice were more susceptible to influenza virus infection, demonstrating that MyD88 signaling is important in resisting primary challenge (75). Although MyD88 signaling was essential for optimal induction of Th1 cells, with Th2 skewing in MyD88 and TLR7 deficient mice (75, 76), Seo et al. found that virus-specific IgG and IgA titers were unaffected and that TLR deficient mice were protected against secondary lethal challenge (75). Other mouse models have found that, despite elevated hemagglutinin inhibition (HI) titers and protective immunity against rechallenge, *tlr7*^−/−^ *myd88*^−/−^ mice demonstrate impaired B cell responses (77). Indeed, TLR7 stimulation has proven critical for isotype class switching in influenza infection (69) and augments antibody secreting cell (ASC) differentiation (78). Taken together this evidence indicates that TLR7 plays an important role in the primary response to influenza, but is not essential to the development of a memory response. In an interesting caveat to this, TLR7 signaling appears to be crucial to the development of protective HI antibodies targeting the pandemic H1N1 (pH1N1) 2009 split vaccine (77), suggesting that the context (infection versus vaccination) in which PRRs recognize viral RNA directs their relative importance. In RSV, TLR7 appears to have an important role in promoting a non-pathogenic T cell response to infection. Engagement of this ligand elicits the IL-12 and type I IFN production required for Th1-type responses, and may skew immunity away from a harmful Th2 response (66, 70, 71). The absence of TLR7 expression in *tlr7*^−/−^ mice does not affect the clearance of RSV from infected animals, but does promote the release of mucogenic cytokines IL-4, IL-13, and IL-17, and preferentially activating IL-23, which is associated with an immunopathogenic Th17 response to RSV in the lungs (79). TLR7 has also proven to have an important role in the humoral response to viruses, inducing B cells to release antigen specific antibodies following sublethal doses of influenza virus (69, 77, 80).

In common with TLR7, the remaining “viral-sensing” intracellular receptors, TLR8 and 9, both initiate proinflammatory cytokine release through the MyD88 signaling pathway. TLR8, which is stimulated by the presence of ssRNA in monocytes and macrophages, induces production of IL-12, proinflammatory IL-6, tumor necrosis factor (TNF), chemokine (C-C motif) ligand/monocyte chemoattractant protein CCL2/MCP-1, and CXCL8/IL-8 but not IFNα (81–83). TLR9, which recognizes unmethylated CpG repeats within DCs, is a potent inducer of proinflammatory and Th1 responses, and upregulates the costimulatory molecules CD80/86. The action of these receptors plays a role in innate immunity and signaling, the impact of which upon viral replication and dissemination has only recently begun to be investigated. During influenza infection increased TLR7/8/9 expression is known to correlate with elevated production of Th1-related cytokines IFN-γ, CXCL10/IFN-γ induced protein 10 and CXCL9/monokine induced by IFN-γ (MIG). The administration of CpG as a TLR9 ligand during exposure to purified RSV F or G proteins or killed bovine RSV (BRSV) also results in Th1-associated immune responses (84–88). Mucosal administration of a TLR9 ligand CpG oligodeoxynucleotides/L18-muramyl dipeptide (CpG ODN/L18-MDP) with inactivated RSV viral particles potently activates NF-κB, shifting cellular immune responses toward a dominant IFN-γ-producing Th1-type response (89). Furthermore, mucosal immunization of BALB/c mice with inactivated RSV and TLR9 ligands induce local IgA responses and Th1-associated IgG2a high-affinity antibody responses (89). It is notable that the engagement of TLR9-induced signaling pathways during FI-RSV immunization is even capable of ameliorating vaccine-enhanced disease (90). TLR9 polymorphisms have also been associated with the risk of developing RSV linked post-bronchiolitis wheeze (91). Thus, it is likely that appropriate TLR-signaling will be required for an effective early vaccine-induced response.

#### Extracellular TLRs in RSV

The extracellular receptors TLR2, TLR4, and TLR6 are commonly recognized as bacterial sensors, given that they recognize lipoproteins, peptidoglycans and bacterial cell-surface lipopolysaccharides, respectively. Nevertheless, these TLRs, which signal through the MyD88 pathway, have been found to play an important role in governing the immune response to RSV. The release of NF-κB-related proinflammatory cytokines from airway epithelial cells is the primary mode of action for TLR2 and TLR6, which do not contribute to the production of type I IFN (92). Engagement of both TLR2 and TLR6 has an effect upon cytokine and chemokine production during RSV infection, and increased viral loads have been observed in mice deficient for these receptors, with impaired neutrophil migration and DC activation in *tlr2*^−/−^ mice (93). The release of proinflammatory cytokines in response to heat-inactivated RSV exposure can also be inhibited by TLR2 blocking antibodies (94). The pathogenic effect is compounded in MyD88^−/−^ mice, as multiple TLR pathways (TLR2, TLR4, and TLR7) are abrogated, leading to increased Th2 cytokine release, mucus hypersecretion, and eosinophilia (66).

However, it is primarily TLR4 which has drawn attention for its interaction with F protein during the early stages of RSV infection (93). Indeed, due to its extracellular nature it is difficult to escape the idea that TLR4 may be the first PRR activated during RSV infection, providing an early innate activation signal sensing infection. TLR4 activation has been associated with RSV clearance in several studies (95–98) and severe RSV disease in infants has been linked to TLR4 polymorphisms (99). In the cotton rat model, inclusion of the adjuvant monophosphoryl lipid A (MPL), which is a partial TLR4 agonist, in an FI-RSV formulation ameliorated the lung pathology associated with vaccine-enhanced disease. This was associated with a reduction in the levels of Th1- and Th2-type cytokines and chemokines normally released following RSV challenge (86, 100). This suggests that while TLR4 engagement is important in mounting an early antiviral response to RSV, it is also capable of provoking complex dysregulation of multiple cytokines. It is therefore not surprising that the RSV G protein may have evolved to target TLR2, 4, and 9 expression in human monocytes, suppressing innate immune responses through multiple downstream mechanisms (101, 102). However, conflicting studies have also suggested that depletion of TLR4 does not impact RSV clearance, nor its infiltration of pulmonary inflammatory cells (103). TLR4 has also been linked to the induction of acute lung injury (ALI) during influenza infection, with exposure to influenza derived antigens inducing nicotinamide adenine dinucleotide phosphate oxidase-dependent production of reactive oxygen species which generate oxidized host phospholipids. These were found to include the oxidized 1-palmitoyl-2-arachidonoyl-phosphaticylcholine, a potent activator of the TLR4-TRIF signaling pathway, which triggers production of proinflammatory cytokines, such as IL-6, by pulmonary macrophages, leading to lung injury (104). Abrogation of TLR4 expression, either through administration of a therapeutic antagonist or use of *tlr4*^−/−^ murine strains, provided protection against virally induced lung injury in mouse models of influenza infection (104–106). Nevertheless, it is likely that, unlike RSV which is recognized by both intracellular and extracellular TLRs, pathogen sensing of influenza is predominantly mediated by intracellular TLRs.

#### Intracellular Sensing by RIG-I-Like Receptors and Innate IFNs

The RIG-I like receptor (RLR) family represent an important additional viral recognition pathway which governs type I IFN production in infected cells. Within the cytosol of the host cell a 5′ triphosphate dsRNA “panhandle” is formed by the conserved 5′ and 3′ end sequences of replicating viral ssRNA. This is detected by one of the RLR helicases, RIG-I (107, 108). RIG-I and melanoma differentiation-associated protein 5 (MDA5) (triggered by longer dsRNAs) both possess caspase activation and recruitment (CARD) domains. MDA5 predominantly senses positive-strand RNA, but can also be triggered by certain negative-strand RNA viruses, such as members of the Paramyxoviridae family (109) and influenza viruses (110). The CARD domains are lacked by the final RLR family member, the laboratory of genetics and physiology 2 (LGP2) molecule. The exact role of LGP2 in antiviral signaling has not yet been fully elucidated, as it has both negative and positive regulatory functions, depending upon the signaling context (111). These adaptor proteins interact with the mitochondrial antiviral-signaling protein (MAVS) or IFN-β promoter stimulator 1 (IPS-1) molecules and initiate signaling transduction cascades that leads to activation of downstream NF-κB and IRF3 pathways (112, 113). This stimulates the induction of type I IFNs. In the case of IFNβ this leads to transcriptional activation of numerous IFN-stimulated genes (ISGs), the most important of which are the IFN-induced transmembrane protein (IFITM) family, the myxovirus resistance proteins (Mx), the serine/threonine kinase protein kinase R (PKR), and the 2′–5′-oligoadenylate synthetase/RNase L system. The IFITM family member IFITM3 has recently been identified as an important receptor in antiviral response against influenza, as it blocks viral entry by impairing virus-host cell membrane fusion, impacting viral susceptibility (114, 115). Both IFITM3 and IFITM1 show a similar impact upon RSV activity, suppressing early viral entry, with IFITM3 in particular delaying the phosphorylation of IRF3 by sequestering RSV in vesicular compartments (116). The Mx family of large guanosine-5′-triphosphate hydrolase enzymes represents another important antiviral factor acting against RNA viruses. In human columnar epithelial cells, the Mx proteins interfere with nuclear transport of viral nucleocapsids to impair influenza transcription and replication (117, 118). The evolutionary pressure exerted by these proteins is capable of causing adaptive mutations in NP which have been observed in pandemic influenza A strains (119).

Given that it is widely conserved across vertebrates, the RLR family clearly plays an important role in sensing and responding to viral infections. An instructive exception to this is demonstrated by the lack of RIG-I receptors within chickens, which has been posited as an explanation for the increased susceptibility of chickens to avian influenza strains, compared to naturally resistant waterfowl such as ducks, which possess a fully functional RIG-I signaling pathway (120, 121). Type I IFNs remain important in chicken antiviral immunity and recent research suggests that the role of sensing influenza infection is assumed by MDA5 in these animals (121). Indeed, animal models, which have long been used as surrogates for evaluating human influenza and RSV infection, show many such unique features, which can unfortunately complicate extrapolation of host and viral factors involved in the immune response to humans (122). This can be observed in the Mx family, as many of the inbred mouse strains used in research, such as C57BL/6 and BALB/c, carry defective genes for Mx1 and Mx2. This has led to a complex picture of MyD88-dependent immunity which long underestimated the role of pDCs in pulmonary defense (123, 124). Mice have been broadly employed to study host-genetic determinants and have helped to identify many host genetic candidates of the genetic host regions involved in antiviral protection (125, 126). However, these animals remain a poor model for respiratory virus transmission, in contrast to which the ferret model is better suited for studying both the pathogenicity and transmissibility of human respiratory viruses (127), despite suffering from a lack of specialized reagents. It is only recently that physiologically relevant human challenge studies have allowed researchers to elucidate in detail the course of disease within immune compartments such as the lung (128, 129). Further systems biology analysis of mucosal tissues obtained during *in vivo* respiratory viral infection will be critical for better understanding of these sensors in humans.

#### Evasion of PRR Recognition and Signaling by RSV and Influenza

The importance of PRRs in the antiviral response and their likely early evolutionary emergence as mediators of protection mean that both RSV and influenza have evolved potent immunomodulatory mechanisms to overcome these pathways, often in a highly species-specific manner (130, 131). During early influenza infection this takes the form of concealing the dsRNA “panhandle” recognized by RIG-I through the action of the nucleocapsid polymerase complex (132). Avian adapted strains which have a reduced PB2-NP affinity are consequently sensed more effectively in cells possessing RIG-I (133). The RSV leader sequence performs a similar function, utilizing the host cellular RNA-binding protein La to inhibit the early RIG-I binding and thus detection of RSV (134). The non-structural proteins derived from both viruses impact the innate immune signaling at multiple points, interacting with the ubiquitin E3 ligases, Tripartite motif-containing protein 25 and Riplet, to prevent RIG-I ubiquitination (135, 136). Influenza NS1 prevents the activation of PKR and the nuclear translocation of transcription factors NF-κB and IRF3 to suppress type I IFN synthesis (131, 137–144). In addition, recent evidence has suggested that influenza HA also targets the positive feedback loop of IFN upregulation by interfering with the IFN receptor (145). Zhang et al. also found that RSV-derived NS1 specifically inhibits IFN-β production by decreasing RIG-I interaction with MAVS (116). The NS2 protein of RSV also inhibits RIG-I activation of IFN promoters by binding to the N-terminal CARD domains of RIG-I and inhibiting MAVS interactions (131). In addition, NS2 induces degradation of the signal transducer and activator of transcription 2 (STAT2) signaling pathways involved in MAVS activation (MAVS) (131, 146). RSV strains deficient in NS1 and NS2 expression show incomplete suppression of type I IFN production during human macrophage and epithelial cell infection, leading to a diminished ability to replicate *in vivo*. Thus respiratory viruses rely to a great extent upon these immunomodulatory proteins to evade the early antiviral response and establish infection. Whether these mechanisms are more potent in RSV than influenza and have a greater impact on downstream impairment of host immunity is difficult to test but they certainly contribute to an immediate failure to control RSV infection as well as probably the inability to elicit long-term protection.

#### Plasmacytoid DCs in RSV and Influenza

Another clue to the reasons for divergence in immunity against the two viruses can be found in the response of neonates and previously uninfected infants to their first encounter with RSV. Until recently, in both murine influenza and RSV, the infiltration of pDCs was thought to play the major role in type I IFN-dependent virus control (147, 148). *In vitro*, while pDCs from adults are capable of responding to cytosolic delivery of RSV-derived dsRNA in a RIG-I–dependent manner (149) by producing type I IFN independent of endosomal TLR activation and PKR (150), similar responses are profoundly impaired in neonates (151). In neonatal mice especially, the limited pDC function and associated type I IFN deficit contribute to Th2-skewed immunopathology in RSV infection (152). Marr et al. not only found that RSV-related IFN-α release is impaired in neonates, but also that peripheral blood cells from children aged between 12 months and 5 years show similarly reduced responsiveness to RSV and synthetic 5′ppp-dsRNA RIG-I agonists (151). This is less clear *in vivo*, where expression levels of MDA-5, RIG-I, TLR7 and TLR8 were all highly elevated in the respiratory tract of infants with bronchiolitis (153). This may have been partially confounded by other respiratory viruses being responsible for bronchiolitis in some of these infants, activating alternative pathways. Plasmacytoid DCs primarily sense ssRNA viruses, including influenza, through the endosomal TLR7/8 pathway (66, 101). In the case of RSV, endosomal maturation and acidification is not required for viral fusion, which may lead to bypassing of the activation of such receptors entirely (154, 155). In contrast, exposure to a low endosomal pH is necessary for the conformational changes that trigger the release of influenza viral ribonucleoproteins from the M1 protein, and subsequent viral-endosomal fusion (156). A fusion pore is then formed, through which viral RNA exits the endosome to the cytosol and then on to the nucleus, where viral replication and mRNA transcription occurs (122, 156, 157). It is possible that pDC exploit this requirement to enter the acidified compartment by initiating proteolytic cleavage and TLR activation. Work in mice lacking asparagine endopeptidase suggests that peptidase-dependent activation of TLR7 is essential for cross priming of CD8^+^ T cells in influenza infection (70, 158). In comparison, RSV not only evades recognition by pDC TLRs, but is capable of abolishing TLR7 and TLR9-dependent IFN-inducing pathways in pDC (150, 154). This difference in the routes taken by the two viruses through the host cells and therefore which PRRs are differentially activated may represent a key divergence. Recent studies have suggested that, *in vivo*, the primary source of type 1 IFN is AMs and not pDCs (see below). It is unclear whether differences in mechanisms of viral entry play a role in this dichotomy.

#### NLRs and Inflammasome Activation by RSV

The cytosolic receptor NLR family consists of members NOD1, NOD2, and NACHT, LRR and PYD domains-containing protein 3 (NALP3, also known as cryopyrin), which are capable of recognizing PAMPs and contributing to the immune response in collaboration with membrane-bound TLRs (159). Conventionally these receptors are activated by molecules, such as muramyl dipeptide, which are produced during the degradation of bacterial peptidoglycan, inducing transcription of immune response genes through NF-κB and mitogen-activated protein kinase signaling pathways. Recent studies have indicated that NOD2 may play a role in sensing viral ssRNA, facilitating MAVS-dependent IRF3 activation and type I IFN production in RSV infection (160). However, it is cryopyrin (encoded by the *Nlrp3* gene), which has garnered attention for its antiviral activity *via* formation of the inflammasome, a caspase-1 activating molecular complex (161). Upon exposure to virally derived ssRNA the NALP inflammasome recruits ASC (apoptosis-associated speck-like protein containing a CARD) and procaspase-1, which is then activated by autocatalytic cleavage to caspase-1. This catalyzes proteolytic processing of pro-IL-1β, pro-IL-18, and pro-IL-33 into active proinflammatory cytokines.

In RSV infection, the SH protein accumulates in lipid rafts of the Golgi apparatus, acting as a viroporin to enhance membrane permeability and promote the entry of ions and small molecules into host cells. This enhances ion-sensitive transcriptional factors promoting viral replication and has also been implicated in NLRP3 inflammasome activation (162, 163). Depletion of SH leads to defective NLRP3/ASC inflammasome activation and IL-1β secretion (163). The M2 protein of influenza also acts as a viroporin, promoting viral uncoating within endosomes and stimulating the activation of the NLRP3 inflammasome (164). *Nlrp3*^−/−^ and *Casp1*^−/−^ mice were more susceptible to influenza infection, with decreased neutrophil and monocyte recruitment and an associated reduction in cytokine and chemokine production (165). This did not, however, translate into an effect on induction of the adaptive immune response. Ichinohe et al. expanded upon this and found that ASC and caspase-1, but not NLRP3, were required for the induction of CD4^+^ and CD8^+^ T cell responses, mucosal IgA secretion and systemic IgG responses (166). Influenza has therefore evolved several mechanisms to evade the activation of the NLRP3 inflammasome. PB1-F2 translocates into the inner membrane space of the mitochondria, acting to decrease the membrane potential and trigger mitochondria disintegration, which blocks formation of the inflammasome. Partial deletion of the amino-terminal RNA-binding domain of NS1 from influenza A rescues the production of IL-1β and IL-18 during infection, suggesting that this domain antagonizes the activation of caspase 1 (167). Intriguingly, Goritzka et al. recently showed that while early lung infiltration of immune cells and levels of proinflammatory mediators were abrogated in *Myd88*/*Trif*/*Mavs*^−/−^ mice (which lack TLR, RLR, and IL-1R signaling), there was no subsequent effect upon the induction of RSV-specific CD8^+^ T cells (168). This implies that caspase-1 and TLR7 are essential to the development of adaptive immune responses to influenza virus, while the RIG-I pathway is more important in RSV but that redundant downstream pathways exist to induce cell-mediated immunity even in its absence.

#### Production of Innate IFNs by DCs and AMs

As discussed earlier, the pDC subset, which express TLRs at a high level, is known to be a potent producer of type I IFNs crucial to antiviral defense, and as such both RSV and influenza have developed mechanisms, such as NS directed antagonism to subvert this immunity (169, 170). This allows the viruses to act upon pDC directed immunity; during the acute phase of infection, patients hospitalized with H1N1 during the 2009 pandemic showed depletion of pDCs, as well as myeloid DCs (mDCs), with pDC counts remaining persistently low for up to 16 weeks (171). The consequences of this are seen in mouse models, which indicate that pDC depletion reduces influenza virus-specific serum antibodies during convalescence (172, 173). The engagement of pDCs in influenza infection, particularly involving TLR7 stimulation, leads not only to induction of type 1 IFNs, Th1 and cytotoxic responses, but also enhances B cell expansion and differentiation into CD27^high^ plasmablasts (174). However, it is clear that pDCs are not essential for the effective induction of CD8^+^ T cell responses or viral clearance (175), and may contribute to immune pathology (176).

Research into the effect of RSV upon pDCs has revealed a contradictory picture of immunity; work in mice has indicated that RSV infection inhibits the ability of lung resident pDCs to respond to TLR activation, reducing IFNα, IL-6, TNF-α, CCL2, CCL3, and CCL4 production, and impairing antigen presentation to T cells (169). This is supported by work in human cord blood derived pDCs exposed to RSV infection, which show reduced IFNα production (151). Conversely, human peripheral pDCs are capable of producing IFNα upon *in vitro* stimulation with RSV (177). While depletion of pDC, in the lungs of mice, affected IFNα levels following influenza, this was not the case with RSV (178), suggesting that pDCs play a less important role in type 1 IFN production during RSV infection than in influenza.

Previous murine studies have implicated AMs, rather than DCs, as major producers of IFNα following RSV infection (172). Monocyte-derived AMs, which make up 95% of all leukocytes found in the airways, are important in antiviral defense, especially within the lower respiratory tract and orchestrate RSV immunity through type I IFN production (173). However, type I IFN deficient murine AMs are still capable of controlling RSV replication (179), suggesting that this may not be their sole function. In concert with studies which throw light onto the involvement of TLR signaling in RSV immunity, these results illustrate the difficulty in extrapolating from mouse models of infection to humans and indicate that peripheral and lung derived pDCs may differ markedly in their function and response to the same viral pathogens.

### ILCs at the Interface with Adaptive Immunity

Innate lymphoid cells are a recently described family of effector cells which bridge innate and adaptive immune systems as well as having a crucial role in mucosal immunity. The prototypic group 1 ILC members, the natural killer (NK) cells, are the only currently known cytotoxic cells within the group and there is accumulating evidence that they have a role in antiviral immunity within the lung. These cells, which develop under the influence of IL-15 released by virally infected bronchial epithelial cells, clearly must achieve a delicate balance in influenza and RSV infection. Numerous studies have highlighted the role NK cells play in both viral clearance and in the exacerbation of virus-induced lung pathology. Mouse models of influenza infection have shown that the presence of NKp46^+^ cells are important in the control of infection (180), with the peak of the antiviral response governed by NK cells displaying an activated phenotype. Human studies have also demonstrated that severe influenza infection is associated with diminished frequencies of circulatory NK cells (181, 182). Welliver et al. found extremely low levels of CD8^+^ T cells and NK cells within pulmonary tissues taken from infants with fatal RSV and influenza LRTI (183). Indeed, these infections were characterized by uncontrolled viral replication in individuals with an inadequate NK response, possibly suggesting a vital antiviral role for NK cells within respiratory tissues. Furthermore, a clue to the lethality of H5N1 influenza strains may be found in their avoidance of NK cell control. The HA protein of human and swine influenza binds to 2,6-linked sialic acid residues on the NK cell receptor, inducing NKp46-mediated killing, while H5N1 (which binds preferentially to 2,3-linked residues), only initiates targeted cell death during human infection following activation of both NKp46 and NKG2D (184, 185). This impaired NK cell activation may help to explain the differential pathogenicity seen in the human and avian strains.

With both viruses, NK cell-derived IFNγ has an important function in the protective response, although the signaling pathways are not yet clearly understood. In the case of RSV, NK cell depleted mice have been found to produce lower levels of IFNγ, with early IFNγ release promoting the development of a Th1 type response. Ordinarily this suppresses production of IL-25, which can drive the development of a Th2 polarized response, characterized by IL-4, IL-13 and mucus production (186). During influenza infection NK cell-derived IFNγ is also required for optimal cytotoxic T lymphocyte (CTL) function and recall responses are augmented by NK cell IFNγ production (187). It is therefore unsurprising that both viruses have developed methods of subverting the NK response, with the G protein of RSV displaying a capacity for CX3C chemokine mimicry and acting as an antagonist to inhibit the trafficking of CX3CR1^+^ NK cells (188). Influenza virus meanwhile, affects killer cell immunoglobulin-like receptors (KIR)/HLA-C interactions, increasing the binding of inhibitory receptors KIR2DL1 and the leukocyte Ig-like receptor, which act to inhibit NK cell function (189). The immunopathogenic effects of NK cell accumulation and IFNγ release within delicate lung tissues are well known in both influenza and RSV infection and may go some way to explaining the increased survival and lowered morbidity seen in NK cell depleted mice infected with influenza (190). This is echoed by a reduction in ALI and morbidity (191) observed in NK cell depleted mice infected with RSV.

Apart from ILC1s, increasing evidence indicates that the other predominant innate effectors involved in respiratory viral infections are the ILC2s. Recent work in mouse models has demonstrated that during the early stages of RSV infection the IL-13 producing ILC2 subset are activated (192). If this finding is emulated in humans, it may be one mechanism by which type 2 immunopathology is promoted in some individuals. The dysregulation of the antiviral immune response can also be seen in influenza infection, in which IL-33 driven secretion of IL-13 by ILC2 cells contributes to airway hyperactivity and may exacerbate disease in asthmatic individuals (193). ILC2s are also the primary source of IL-5, another type 2 cytokine induced by IL-33 secretion, which has been shown to promote the accumulation of eosinophils and the exacerbation of disease in influenza infected lungs of mice (194), and these lung resident ILC2s contribute to the induction of an immunopathogenic type 2 response in RSV and influenza infections. This picture is somewhat complicated by emerging evidence that ILC subsets exhibit plasticity depending upon the inflammatory milieu. ILC3 cells are known to differentiate into ILC1 cells (and vice versa), and TLR2 promotes the production of IL-5 and IL-13 by ILC3 cells, suggesting that they are capable of differentiating into ILC2 cells. The ILC3 subset, which express retinoid-related orphan receptor γt and produce IL-17A/F and IL-22 has not been previously implicated in antiviral responses. However, work by Stier et al. has found that during RSV infection, reduced numbers of antiviral IFN-γ^+^ ILC1 and increased numbers of pathogenic IL-5^+^ and IL-13^+^ ILC2 and IL-17A^+^ ILC3 accumulate in the lungs of STAT1-deficient mice (195). This raises the possibility that the inflammatory ILC3 subset may play a role in virally induced lung pathology. Alongside the newly described ILC regulatory subset, it remains to be seen, however, whether ILC3 cells have a functional role in the antiviral response.

The inflammatory milieu within the lungs during viral infections is heavily composed of neutrophils. These short-lived, terminally differentiated, phagocytic cells have been implicated in the early response to RSV, especially in infants which go on to develop bronchiolitis (196). Their recruitment to sites of infection within the mucosal tissues is associated with viral killing by oxygen dependent and independent mechanisms. In addition to this secretion of antimicrobial products through degranulation, activated neutrophils also release neutrophil extracellular traps (NETs), which consist of chromatin and granule proteins such as elastase. Neutrophil-derived elastase and chemoattactants such as IL-8 are elevated during initial RSV infection, and neutrophils are capable of ameliorating disease during influenza infection (197, 198). During the course of infection with both viruses an influx of neutrophils can stimulate antiviral IFNγ release by CD8^+^ T cells (199, 200). In mouse models of influenza infection, administration of the mAb 1A8 (which specifically targets the Ly6G antigen expressed by neutrophils), enhances viral replication and exacerbates pulmonary inflammation, edema, and respiratory dysfunction (201). Depletion of neutrophils with 1A8 also impairs the cytotoxic function and cytokine release of influenza specific pulmonary CD8^+^ T cells (202), and can lead to increased susceptibility to lethal challenge with virulent influenza strains (198). However, contradictory evidence suggests that excessive infiltration of neutrophils into the airways is associated with a fatal outcome in H7N9 and H1N1 infected patients, with NETs playing a key role in influenza induced lung damage (203). The importance of neutrophil infiltration and formation of NETs in ALI has also been observed in mouse models of sublethal influenza challenge. Following administration of 1A8 mAb, mice exposed to H1N1 on a PR8 backbone strain demonstrated milder lung pathology than macrophage depleted mice (204). Research by Brandes et al. suggests that neutrophil infiltration establishes a damaging feed-forward loop which is dependent upon the viral strain and inoculating dose (205), which may go some way to explaining the discrepancies observed in the pathogenic role neutrophils play during influenza infection. It appears that while recruitment of these destructive cells to the delicate tissue of the lung may be crucial in early clearance of viral particles, their proinflammatory nature can also cause substantial immune pathology.

### Antigen Presentation and Induction of Proinflammatory Cytokines by DCs

Dendritic cells play a crucial role in linking the innate and adaptive immune systems, especially at mucosal surfaces. The majority of immature DCs fulfill a sentinel function in the periphery, including the respiratory system. Here, they may remain for up to two weeks before leaving tissues *via* the lymphatics, although the turnover of DCs in the mucosa is much faster. Engagement of PRRs expressed by DCs leads to the upregulation of costimulatory molecules, and the generation of proinflammatory cytokines, such as IL-6, IL-12, and TNF, which trigger a maturation program determining how a naive T cell responds to antigen stimulation. The wide range of pulmonary DC subsets fulfill distinct, occasionally overlapping functions, influencing viral clearance from the lung and induction of T cell responses. Following PRR stimulation, DCs downregulate the expression of tissue retaining chemokine receptors, CXCR1, CCR1, CCR2, and CCR5, and upregulate CCR7 and CD11c, allowing the DC to home to secondary lymphoid organs. Within the lymph node or spleen the mature DC, which has massively upregulated the expression of both peptide-loaded major histocompatibility complex (MHC) and costimulatory molecules at the cell surface, is capable presenting antigen to cognate T cells. Murine lung DCs can be divided into CD103^+^ conventional DCs (cDCs) and CD11b^+^ cDCs (in humans these correspond, respectively, to the CD141^+^ and CD1c^+^ DC subsets), and plasmacytoid DCs (pDCs). Monocyte-derived DCs are also generated in the lung during inflammatory episodes.

Influenza and RSV infection have both been reported to cause CD103^+^ cDCs to migrate from the intraepithelial basement layer to draining mediastinal lymph nodes for antigen presentation to naive T cells (175, 206). However, severe influenza infection particularly leads to lymph node accumulation of CD11b^+^ cDCs in the lymph nodes, with these DC subsets dominating antigen presentation at the peak of infection (207). As priming during early infection is characterized by a more even balance of CD11b^+^ and CD103^+^, it is unclear whether severity of infection, inoculating dose or temporal kinetics govern the subsets activated. In the context of viral immunity it is important to note that these DCs tend to differ in their antigen presentation. CD11b^+^ cDCs readily present viral antigens to CD4^+^ T cells through MHC class II, promoting both Th2 and Th17 responses. CD103^+^ cDCs, meanwhile are capable of biasing toward a Th1 response, but also specialize in the cross-presentation of antigen through MHC class I pathways, driving CD8^+^ T cell priming. This is further complicated by studies indicating that differential expression of CD24 by CD103^+^ DCs and CD11b^+^ DCs lead to preferential induction of effector and central memory CD8^+^ T cells, respectively (208). Bearing this in mind, it is unsurprising that depletion of CD11c^+^ cDCs results in the impaired development of influenza virus-specific CD8^+^ T cells in the mouse model (175, 209),.

Significantly, studies in neonatal mice have described a fundamental shift in the balance of cDC subsets in early life. While both CD103^+^ and CD11b^+^ cDCs are substantially less effective in infants than adults, a functionally limited CD103^+^ population dominates the response in favor of a diminished CD11b^+^ cDC subset in early life (210). In mice, this shift in cDC subsets influences the CD8^+^ T cell epitope hierarchy during RSV infection, dramatically changing the immunodominant epitopes between infancy and adulthood (210, 211). Recent work by Ruckwardt et al. suggests in the neonatal mouse model that both RSV and influenza infection lead to the production of a distinct CD103^low^ population which predominates over CD103^high^ in early life. These express lower levels of lineage-defining markers and costimulatory molecules, as well as more limited antigen uptake and processing capabilities (211). This highlights the complex and subtle changes undergone by the immune system at different ages, confounding our understanding of antiviral immunity in patients.

## Differential Induction of Long-Term Humoral Immunity by Influenza and RSV

Natural infection by influenza is known to induce long-lived high affinity antibodies that are associated with complete protection from symptomatic infection for many years (212). However, the defining characteristic of influenza vaccination is the requirement for reformulation on a regular basis to match predominant circulating strains. This is due to the majority of human antibodies induced by influenza being directed toward the immunodominant globular head domain of HA. Following infection, high levels of strain-specific anti-HA antibodies confer long-lasting protection that can endure for many years, but rapid accumulation of mutations in the globular HA head leads to antigenic escape. Recent work to develop a cross-strain protective vaccine has therefore focused on inducing antibodies against the more conserved HA stalk. Mouse studies have demonstrated that sequential exposure to divergent influenza strains can trigger the production of broadly neutralizing anti-stalk antibodies (213). In humans, infection with pH1N1 boosted the production of HA stalk antibodies and these neutralizing antibodies could in principle be replicated by vaccination (214). However, while neutralizing antibodies are understood to correlate with protection against influenza infection, it has been suggested that heterosubtypic stalk-binding mAbs may be less effective and display limited activity against cell-bound viruses (215). Nevertheless, conflicting evidence has revealed that not only may these antibodies actually outperform conventional neutralizing antibodies within a more physiologically relevant polyclonal condition (216), but also that *in vivo* these antibodies are able to activate antibody-dependent cellular cytotoxicity which correlates with enhanced antiviral protection (217).

Historically vaccine efforts against RSV have also focused on the induction of neutralizing antibody titers, and an association between systemic antibody levels and protection from severe disease has been shown both in hospitalized patients and in animal models (218). Indeed, the efficacy of the mAb palivizumab in reducing RSV-related hospitalization in high-risk infants proves the efficacy of serum IgG alone in preventing RSV LRTI (219). However, it has long been known that while higher levels of neutralizing antibody and F protein-specific IgG do correlate with protection from severe disease, it is still possible to experimentally re-infect adults with RSV regardless of their systemic antibody levels (11). This contrasts with influenza, where re-challenge after an interval of as much as 7 years fails to induce any viral replication or symptoms (212), and pH1N1 antibodies are long-lasting (220). This has led to difficulty in identifying a threshold of protective titers for anti-RSV antibodies. Furthermore, levels of antibody show a precipitous decline post-infection and do not provide incremental protection during subsequent RSV seasons (221).

As respiratory viruses, both RSV and influenza are capable of inducing mucosal IgA, which acts as first-line antiviral defense. Mouse models have confirmed that these antibodies are secreted rapidly in the upper airways following primary infection with RSV (222), and in both viruses secretory IgA (sIgA) has been shown to confer protective immunity in mice (222–224). In humans the role of sIgA in protection is relatively poorly understood. However, recent RSV challenge studies have revealed that, while serum antibody does not significantly affect the likelihood of infection, nasal anti-RSV and anti-F protein IgA titers are significantly higher in individuals who are resistant to experimental challenge (225). This is supported by findings by Bagga et al. confirming that pre-existing nasal IgA is a predictor of lower infectivity and viral replication, although in this model, serum neutralizing antibody titers played a greater role in predicting immunity (226).

Antibody producing B cells can be generated in a T cell dependent or independent manner. The majority of IgA^+^ memory B cells (MBC) and long-lived IgA^+^ plasma cells develop within the germinal centers (GC) of peripheral lymphoid organs, undergoing affinity maturation and somatic hypermutation, predominantly requiring T-cell help *via* CD40L and transforming growth factor (TGF) β1, although T-cell independent B-cell class switching has been observed in the GC, mediated by follicular DCs (fDC). DCs are derived from mesenchymal rather than hematopoietic origin, and unlike pDCs and mDCs they do not express MHC molecules at the cell surface; instead antigens complexed to antibodies or complement are displayed at the cell surface for recognition by B lymphocytes. These antigen presenting cells (APCs) are a key component in the generation of long-lived MBC and plasma cells, but mature fDC networks are absent in early life, which may limit responses of T cell dependent ASCs. MBCs and plasma cells have distinct phenotypic characteristics, which may be impacted by early life experiences with enduring consequences upon immune responses. Human challenge models have demonstrated that while IgG^+^ MBC frequencies increase following both RSV and influenza infection these cells did not influence the outcome of viral challenge (225). There was however a profound defect in the induction of RSV- and F-specific IgA-producing MBCs which showed no detectable increase in frequency following RSV infection (225). This can be contrasted with individuals naturally infected during the 1918 influenza pandemic, who were found to have functional strain specific MBCs circulating up to 70 years following exposure (227) with long-lived plasma cells producing high affinity antibodies against not only 1918 strains, but also contemporary pH1N1 from 2009 (228).

Antigen-specific IgG and IgA producing MBCs also develop at extrafollicular mucosal sites in a T cell dependent or independent manner, involving B cell activating factor of the TNF family (BAFF) and a proliferation-inducing ligand (APRIL), which in influenza infection leads to protection against reinfection with similar strains (229). BAFF protein expression has been found in the upper airways of children with influenza virus, as well as in the lower airways of infants with severe RSV bronchiolitis (230). However, recent work in the closely related BRSV has suggested that the SH protein not only inhibits NF-κB expression in APCs (231), but also blocks phosphorylation of STAT1 thereby inhibiting BAFF expression and potentially interfering with induction of B cell responses. Infection of APCs with an SH deletion mutant led to increased production of BAFF and APRIL. It remains to be seen whether this action of the human RSV SH protein may contribute to the peculiar defect in MBC induction seen in RSV, but not influenza.

## Generating Effective T Cell Help in Influenza

Animal models have clearly shown that both CD4^+^ and CD8^+^ T cells are essential for effective resolution of viral infection and protective memory. Influenza vaccination strategies may therefore be improved by targeting conserved regions of HA and NA antigens for presentation by MHC molecules to expand the CD4^+^ T cell repertoire. This is an attractive strategy as CD4^+^ T cells are capable of supporting both humoral responses and enhancing CD8^+^ T cell immunity to generate high affinity and cross-protective responses. The predominant CD4^+^ population capable of inducing the generation of high-affinity, class-switched antibodies are the T follicular helper (TfH) cells which reside within the GC. These cells are involved in the production of IL-2, IL-4, IL-10, TGFβ, and IL-21, which promotes the generation of plasma cells and MBCs (232). TGFβ and IL-21 in particular, have been found to promote class-switching and stimulate the generation of IgA^+^ plasmablasts (233). These cytokines also act to downregulate CXCR5 and upregulate CCR10 on plasmablasts, enhancing migration toward local mucosa. TfH cells expressing CXCR5, programmed cell death protein 1 (PD-1), inducible costimulator (ICOS), CD28, CD40L, the adaptor protein SAP, and the B cell lymphoma 6 (Bcl-6) transcription factor are abundant in the nasopharynx-associated lymphoid tissues during early childhood. Stimulation by live attenuated influenza vaccine induces a marked increase in TfH frequency which correlates with anti-HA IgA IgG and IgM antibodies in human tonsillar cells (234). Work by Leon et al. in mice has furthered our understanding of the GC TfH response to viral antigens, suggesting that FoxP3^+^ Tregs play a critical role in the differentiation of influenza-specific TfH cells by regulating the availability of suppressive IL-2 *in vivo* (235). Miyauchi et al. has also posited that mice deficient in Bcl-6, and consequently TfH cells, are capable of producing low affinity IgG2 neutralizing antibodies, which nevertheless provide protective immunity against lethal challenge with pathogenic H5N1 and pH1N1 strains (236). It is possible, in this case, that the role of promoting B cell proliferation and differentiation into plasma cells was assumed by IL-21 and IFNγ-secreting CXCR3^+^CD4^+^ Th1 cells. The existence of a circulating compartment of TfH cells within the periphery has also been recently demonstrated in humans. This ICOS^+^ PD-1^+^ CXCR3^+^ population correlates with the generation of high-avidity antibodies following influenza vaccination (237, 238).

There is a paucity of work demonstrating the role of CD4^+^ T cells in human RSV. The first work exploring the impact of RSV upon TfH suggested that this virus is capable of upregulating expression of programed death ligand 1 (PD-L1) on murine DCs and B cells to decrease TfH production of IL-21 and expression of IL-21R (239). It is already known from coculture experiments that blocking PD-L1 on RSV-infected bronchial epithelial cells enhances effector-memory and terminally differentiated CD8^+^ T cell secretion of IFNγ, and granzyme B, enhancing viral clearance (240). It is currently not known whether Tregs affect TfH differentiation in response to RSV, with the impact of Tregs during an RSV infection related to their recruitment of activated CTLs into the lungs, increasing viral clearance (241). RSV may also act to inhibit Treg differentiation, stimulating a pathogenic Th17/Th2 response (242, 243). If defects in CD4^+^ T cell help of B cells, such as a dysregulated PD-L1/IL-21 axis or failure to promote class switching to IgA in the secondary lymphoid tissues, can be demonstrated in human RSV infection, this may provide fresh clues regarding mechanisms by which this virus modulates the protective adaptive response.

## Impaired Cell-Mediated Immunity in Human RSV

The protective and pathogenic effects of T cells in respiratory virus infections have been extensively reviewed elsewhere (244–247). Mouse models have shown that both CD4^+^ and CD8^+^ T cells mediate RSV clearance (248), and studies in hospitalized infants have demonstrated a robust antigen-specific CD8^+^ T cell response peaking at convalescence (249, 250). In addition, heterosubtypic immunity against influenza in mice is promoted by crossreactive CD8^+^ T cells (251), while antibody production is impaired in T cell deficient mice (252). In humans, both CD4^+^ and CD8^+^ influenza specific T cells have been shown to correlate with reduced disease severity in experimental challenge and natural infection (129, 253).

However, in human RSV, impairment in T cell function has been implicated in susceptibility to recurrent infection. For example, population-based studies have shown an association between reduction in T cell numbers and function and susceptibility to more severe disease. This is perhaps more obvious in older adults, where an age-related decreases in the numbers of RSV-specific T cells and ratio of CD8^+^/CD4^+^ memory T cells expressing IFNγ is associated with waning immunity observed in elderly adults (254–256). However, confounding difficulties with confounders in natural infection studies have meant that there are also studies which do not show impaired CD8^+^ T cells activation in response to RSV infection in the elderly (257).

Human challenge studies of young adults have more directly demonstrated the limited polyfunctionality of circulating RSV-specific CD8^+^ T cells compared to influenza-specific CD8^+^ T cells from the same individuals (128). During RSV infection cDCs and pDCS are capable of migrating to draining lymph nodes bearing antigens from the lung, but these cells have proven poor inducers of CD4^+^ T cell proliferation (177). *In vitro* studies have also suggested that RSV infection causes suboptimal DC activation and that these DCs are defective in antigen presentation *via* the immune synapse (258). Given that primary expansion, CTL activity and effective long-term memory of antiviral CD8^+^ T cells are all optimally dependent upon CD4^+^ T cell help, this may suggest a mechanism for RSV impaired CD8^+^ T cell function as well as B cell memory. Indeed, in an *in vitro* coculture system, DCs pulsed with recombinant RSV NS1-deletion mutant resulted in an increased activation and proliferation of tissue homing CD103^+^ CD8^+^ T cells and antiviral Th17 cells, suggesting an NS1-mediated mechanism of immune modulation being responsible for CD8^+^ T cell impairment (259). RSV infection of DCs has also been shown to inhibit the production of proinflammatory cytokines, including IFNγ (260). The result, it has been speculated, is suboptimal signaling to T cells and therefore failure to optimally stimulate a full program of differentiation for the generation of long-lived polyfunctional T cells.

In mouse models of influenza Tem cells proliferate in the lungs contributing to heterosubtypic immunity (261, 262). However, Jozwik et al. did find that local virus-specific CD8^+^ T resident memory (Trm) cells in the airway (that express the canonical markers CD103 and CD69) correlated significantly with reduced RSV disease severity, suggesting that while T cell functionality might be impaired, their presence at the site of infection could still lead to improved outcome (128). The role of Trm cells in immunity against respiratory viruses has only recently begun to be unraveled, with these cells differentiating in the lymph node following antigen presentation by CD103^+^ DCs. Given that both RSV and influenza infection promote CD103^low^ DCs in early life, it is tempting to speculate that this represents an attempt by the viruses to subvert Trm production. Recent work by Zens et al. has also shed light upon the inefficient generation of Trm cells observed in early life (263). It appears that the reduced generation of Trm may be a factor intrinsic to infancy, as mouse models of influenza infection and vaccination demonstrate ineffective generation of lung Trms and promotion of long term memory, despite the generation of CD4^+^ and CD8^+^ effector T cells capable of viral clearance (263). In contrast to immunity in adults, in infants the distinct transcriptional profile of these lung-homing T cells favors enhanced T-bet expression and reduced expression of the survival factor CD127, leading to pathogen clearance by terminally differentiated T cells. Furthermore, it is clear from influenza mouse models that CD4^+^ T cell help is required for the promotion of CD103^+^ CD8^+^ Trm cells (264) and so both play an important part in the induction of heterosubtypic immunity (265). Interestingly, unlike most other tissues where Trm cells have been investigated, lung Trm cells are not persistent and undergo attrition over time (265, 266). In view of the work by Jozwik et al. this suggests that while Trm can be important in the early clearance of viruses from the lung, RSV-specific impairment of T cell functionality contributes more rapidly to the downregulation of protective immunity, compared with influenza.

It remains to be seen whether stimulation of Trm cells by influenza or RSV vaccination can enhance protection but if indeed they can, a careful balance is likely to be required. Data from healthy young adults in experimental challenge and natural infection studies as well as in infants with severe RSV and influenza LRTI all suggest that lower CD8^+^ T cell numbers may be associated with more severe disease (183). H owever, induction of T cell immunity in the delicate mucosal structures of the lung has also been associated with greater severity of disease, with a higher frequency of activated T cells in both experimentally infected mice (248, 267) and RSV-infected adults who required hospitalization (257). This dysregulation of the immune system may also be seen in influenza infection with an increased release of proinflammatory cytokines. Thus, cell-mediated immunity is critical in clearing viral infection and coordinating the adaptive immune response as a whole. However, the exact role of each subset, their anatomical location and how they can be induced and maintained at optimum levels must be better understood before harnessing them to improve protection.

## Conclusion

It has long been recognized that influenza and RSV infection lead to highly distinct clinical and immunologic outcomes in humans. While the long-lived antibody and cell-mediated protection conferred by influenza infection is the prototype of a complete and robust antiviral response, immunity against RSV is poor in all settings and unable to fully protect against recurrent symptomatic infection even with an identical strain. It is increasingly clear from natural infection and human challenge studies that the immediate reason for this is impairment in quantity, quality and durability of antibodies, B cells and T cells. However, the underlying mechanisms leading to this remain poorly understood. It seems likely from the wealth of *in vitro* and animal data that both influenza and RSV subvert innate immunity in order to establish infection (Table 1). However, RSV additionally impairs long-term memory generation. The relative contribution of mechanisms such as alteration of the inflammatory environment, IFN inhibition, and impaired antigen presentation is still unclear. By further understanding of how influenza induces robust immunity whilst RSV evades it, a more general understanding of the critical components of an effective immune response is now coming through but many knowledge gaps remain. Development of novel vaccination strategies are likely to require these insights so that immunogenicity can be optimized through avoiding or overcoming pathogen-induced immunomodulatory mechanisms and thus successfully elicit long-term protective immunity.

**Table 1:** Comparative immunity against influenza and respiratory syncytial virus (RSV).

	Influenza	RSV
Clinical outcome of natural infection	Robust strain-specific protection	Recurrent symptomatic infection throughout life
Virology	Highly variable surface glycoproteins Segmented genome	Major surface target F protein highly conserved Non-segmented genome
PRR recognition	TLR 3/7/8/9 sensing RIG-I sensing NLRP3 inflammasome partial evasion	TLR 3/7 sensing TLR 2/4/6 sensing RIG-I less important? NLRP3 inflammasome activation
Primary sources of innate IFN	Plasmacytoid DCs Epithelial cells	Alveolar macrophages Epithelial cells
Suppression of innate IFN	NS1 block PKR	NS1 and NS2 block RIG-I/MAVS interaction NS2 degrades STAT2
Recruitment of immune cells	Th1-promoting environment	CX3C chemokine mimicry dysregulates inflammation
Antibodies	Protective and long-lasting	Protective but short-lived
B cells	Robust IgG^+^ and IgA^+^ MBCs	IgG^+^ MBCs Poor IgA^+^ MBC response Inhibition of BAFF?
T cells	Th1 and CD8^+^ dominated Highly polyfunctional	Th2/Th17 bias in early life Poor polyfunctionality

## Author Contributions

All authors listed have made a substantial, direct, and intellectual contribution to the manuscript and approved it for publication.

## Conflict of Interest Statement

The authors declare that the research was conducted in the absence of any commercial or financial relationships that could be construed as a potential conflict of interest.
